# Improved blood velocity measurements with a hybrid image filtering and iterative Radon transform algorithm

**DOI:** 10.3389/fnins.2013.00106

**Published:** 2013-06-18

**Authors:** Pratik Y. Chhatbar, Prakash Kara

**Affiliations:** Department of Neurosciences, Medical University of South CarolinaCharleston, SC, USA

**Keywords:** blood flow, two-photon imaging, space-time images, line-scan, velocity, Sobel filtering

## Abstract

Neural activity leads to hemodynamic changes which can be detected by functional magnetic resonance imaging (fMRI). The determination of blood flow changes in individual vessels is an important aspect of understanding these hemodynamic signals. Blood flow can be calculated from the measurements of vessel diameter and blood velocity. When using line-scan imaging, the movement of blood in the vessel leads to streaks in space-time images, where streak angle is a function of the blood velocity. A variety of methods have been proposed to determine blood velocity from such space-time image sequences. Of these, the Radon transform is relatively easy to implement and has fast data processing. However, the precision of the velocity measurements is dependent on the number of Radon transforms performed, which creates a trade-off between the processing speed and measurement precision. In addition, factors like image contrast, imaging depth, image acquisition speed, and movement artifacts especially in large mammals, can potentially lead to data acquisition that results in erroneous velocity measurements. Here we show that pre-processing the data with a Sobel filter and iterative application of Radon transforms address these issues and provide more accurate blood velocity measurements. Improved signal quality of the image as a result of Sobel filtering increases the accuracy and the iterative Radon transform offers both increased precision and an order of magnitude faster implementation of velocity measurements. This algorithm does not use *a priori* knowledge of angle information and therefore is sensitive to sudden changes in blood flow. It can be applied on any set of space-time images with red blood cell (RBC) streaks, commonly acquired through line-scan imaging or reconstructed from full-frame, time-lapse images of the vasculature.

## Introduction

The need to understand the relationship between neuronal and vascular activity has become crucial with the rise in the use of functional magnetic resonance imaging (fMRI) in research (Smith, 2012). Two-photon and other imaging techniques like bright field microscopy have provided us with the means to follow changes in neuronal and vascular activity at high spatial and temporal resolution. Neuronal activity can be determined by imaging calcium signals as a surrogate for spiking activity (Tsien, 1988; Stosiek et al., 2003; Ohki et al., 2005; Tian et al., 2009; Akerboom et al., 2013), while blood flow can be calculated from the vessel diameter and blood velocity.

**Figure 1:**
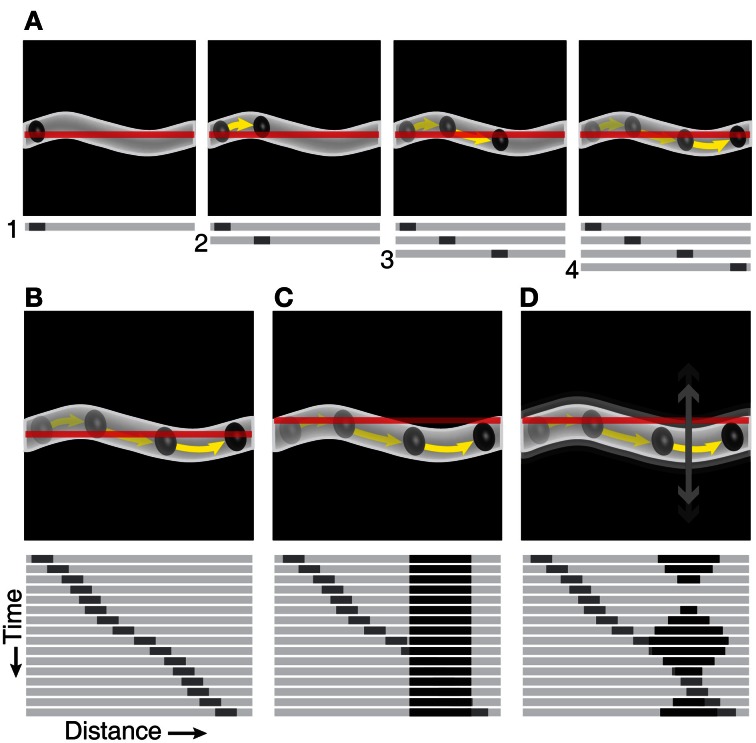
**Schematic of using a line-scan protocol to create space-time images under ideal recording conditions and when artifacts are present. (A)** Full-frame image showing an RBC traveling inside a vessel. The line-scan path is represented as a red line (in top panels) and a stack of sequential line-scans shows the RBC at four different locations in the vessel (bottom panels). **(B)** Repeated line-scans through the identical location in the vessel (top panel) results in a space-time image that has a diagonal RBC streak (bottom panel). **(C)** A line-scan path that goes outside the vessel (top panel) results in a time-invariant artifact, represented as a black vertical band in the line-scan image (bottom panel). **(D)** Movement of the vessel relative to the line-scan path (top panel) results in a time-varying artifact, represented by the variable width of the black band in the line-scan image (bottom panel).

Vessel diameter can be measured by labeling the vessel wall, for instance with an artery-specific dye (Shen et al., 2012), or by labeling the vessel lumen with circulating fluorescein-dextran (Schaffer et al., 2006; Drew et al., 2011). Blood velocity can be measured from the speed of red blood cells (RBCs) inside the vessel by labeling either the plasma or RBCs. Intravascular injection of large dextran-conjugated fluorescent dyes lead to labeling of the plasma. Alternatively, RBC-labeling with smaller lipophilic fluorescent dyes can also be used for studying vascular blood flow (Kamoun et al., 2010). These selective plasma or RBC-labeling methods lead to high contrast between RBCs and plasma, resulting in streaks of RBCs in the space-time images of vessels (Figures 1A,B). A given streak angle depends on the distance traveled by the RBC per unit time. Therefore, blood velocity can be determined from the slope of such streaks.

Calculation of the slope of the RBC streaks has been proposed using many different algorithms including line fitting (Zhang et al., 2005), singular value decomposition (SVD) (Kleinfeld et al., 1998), Radon transform (Drew et al., 2010), and Fourier transform (Autio et al., 2011; Kim et al., 2012). Calculation of velocity using global energy minimization has also been proposed (Deneux et al., 2011). The Radon transform is one of the most powerful blood velocity measurement algorithms because of its easy implementation, accuracy, speed, and robustness. Radon transform calculates the slope from the angle that provides the maximum variance in the projections of the line-scan image at a specified angle space. However, it can still produce erroneous results when the line-scan images have low contrast and/or include artifacts (Figures 1C,D). Also, the precision of the velocity measurements is based on the angle step-size used for the Radon transform. Thus, selecting the number of angle steps to process with the Radon transform involves a trade-off between computation time and the precision of the measured streak angle.

Here we propose an enhanced algorithm that increases the accuracy and speed of velocity measurements: Sobel filtering of the image is followed by iterative application of multiple Radon transforms with increasing angle precision. Contrary to vertical (temporal) or whole-image demeaning, Sobel filtering (Sobel, 1978) efficiently removes the baseline or slow trends from the line-scan image. This translates into more accurate angle (and therefore velocity) measurements from line-scan images. Our development of an iterative Radon transform is built on the idea of adaptive application of Radon transforms with finer angle steps (see Drew et al., 2010 and our Materials and Methods). The first iteration of Radon transforms uses large angle steps across the full range of angles with progressively smaller steps in the subsequent iterations, allowing very precise angle calculations without the trade-off of exceedingly long computation times.

## Materials and methods

### Experimental methods

Long Evans rats (postnatal days 29–48) and cats (postnatal day 28—adult) of both sexes were used to collect images from the primary visual cortex. The Institutional Animal Care and Use Committee of the Medical University of South Carolina approved all animal experiments. Detailed experimental setup, anesthesia, surgical procedures, and drifting grating visual stimulation protocols are described elsewhere (O'Herron et al., 2012; Shen et al., 2012). Briefly, rats were anesthetized with intraperitoneal injection of a fentanyl cocktail (0.04–0.06 mg kg^−1^ fentanyl citrate, 3.75–6.25 mg kg^−1^ midazolam, and 0.19–0.31 mg kg^−1^ dexmedetomidine). Cats were anesthetized with 1–2% isoflurane. After making a 2–3 mm craniotomy and removal of dura, the vasculature was visualized with circulating 2000 kDa Fluorescein Dextran (FD2000S, Sigma), 75 kDa Texas Red Dextran (Life Technologies), or Alexa Fluor 633 Hydrazide (Life Technologies). Imaging was performed with a custom-designed two-photon microscope and a water-immersion objective (Olympus XLUMPLFLN 20×, 1.0 NA or Olympus LUMPlanFI/IR 40×, 0.8 NA). Circulating RBCs were visualized as dark disks against the fluorescent-labeled plasma. These RBCs appear as dark bands or streaks in space-time images. In both rats and cats, movements of the brain were minimized by sealing the craniotomy with agarose and a coverglass (Levy et al., 2012; O'Herron et al., 2012). In cats, a lumbar suspension and pneumothorax were needed to further reduce the brain movements of cardiac and respiratory origin. Typically, brain movement was limited to 1–2 μm in rats and kittens. But in some adult cats, movements of up to 5 μm were present.

### Data collection parameters

Fractional change in blood velocity is best detected when the RBC streak-angle is ~45° (see Equations 16, 17). However, it is not always possible to acquire line-scan images with RBC streaks at this angle because of fast flow and/or relatively slow imaging, e.g., when using galvanometers. In such cases, the line-scan acquisition rate can be increased by imaging a smaller portion of the vessel, and/or by reducing the spatial resolution and pixel dwell-time of the scan, as long as the RBC-plasma contrast is sufficiently high. Additionally, the end-to-start flyback time during scanning can be reduced by combining a low-magnification objective with a compensatory zoom, e.g., 20× objective at zoom 4 instead of 40× objective at zoom 2. We observed that this slight advantage in speed gave superior line-scan acquisitions with a steeper slope of the RBC streaks and more reliable velocity measurements, e.g., in 30–50 μm diameter arterioles that have high blood speeds. Additionally, flyback speeds can be improved by driving the beam steering hardware to the fastest possible speeds.

For a vessel with blood velocity of *v* mm/s captured with line-scan parameters of Δ*x* μm/pixel spatial resolution and Δ*t* ms/line temporal resolution (line-scan time), the angle of the streak θ is,

(1) $$\theta =\text{arctan}\left(\frac{v\Delta t}{\Delta x}\right)$$

The value of θ depends on blood velocity as well as spatial and temporal resolution of the line-scan image. If changing the imaging parameters offers a new angle θ_new_ as a result of new spatial resolution of Δ*x*_new_ μm/pixel and temporal resolution of Δ*t*_new_ ms/line, the relationship between the new and old angles can be shown as,

(2) $$\theta_{\text{new}}=\text{arctan}\left(\frac{\Delta x}{\Delta x_{\text{new}}}\frac{\Delta t_{\text{new}}}{\Delta t}\text{tan}\left(\theta \right)\right)=\text{arctan}\left(\frac{1}{k}\text{tan}\left(\theta \right)\right)$$

Where *k* is defined as,

(3) $$k=\frac{\Delta x_{\text{new}}}{\Delta x}\frac{\Delta t}{\Delta t_{\text{new}}}$$

With the current streak angle θ and the desired streak angle θ_new_, the imaging speed factor *k* can be calculated as,

(4) $$k=\frac{\text{tan}\left(\theta \right)}{\text{tan}\left(\theta_{\text{new}}\right)}$$

**Figure 2:**
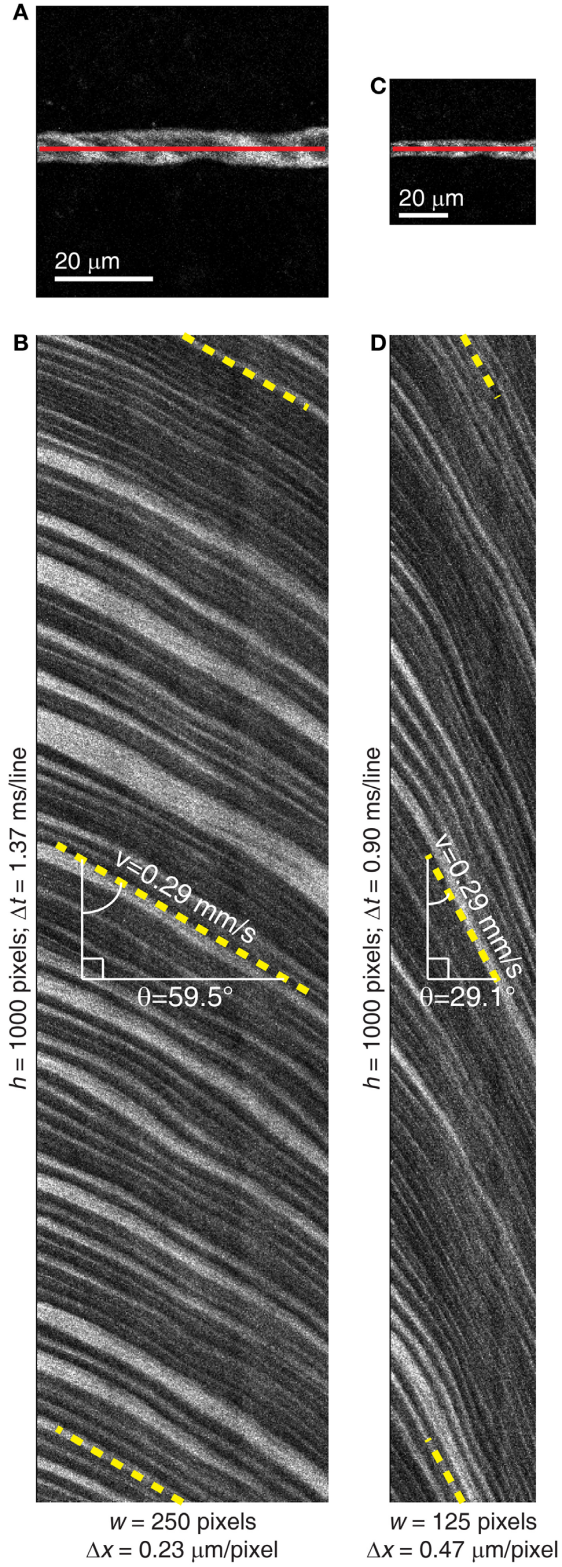
**Change in the pixel resolution leads to change in the RBC streak angle. (A)** Full-frame image of an 8 μm diameter microvessel segment in the rat visual cortex with the line-scan trajectory shown as a red line. **(B)** Space-time image collected from the vessel shown in **(A)**. Line-scan imaging with spatial resolution (Δ*x*) of 0.23 μm/pixel and period (Δ*t*) of 1.37 ms/line was used. Pixel dwell time was 4 μs. The RBC streaks have angle of 59.5° (dashed yellow lines) and blood velocity of 0.29 mm/s. **(C)** Full-frame image of the same microvessel segment as shown in **(A)** but with coarser spatial resolution. **(D)** Space-time image collected with the same line-scan path but with bigger pixel size (Δ*x* = 0.47 μm/pixel) leading to shorter line-scan time (Δ*t* = 0.9 ms/line). This resulted in the RBC streak angle of 29.1° (dashed yellow lines) and blood velocity of 0.29 mm/s. The mean ± SD velocity from the acquired 20 line-scan images was 0.28 ± 0.02 mm/s in **(A)** and 0.29 ± 0.02 mm/s in **(C)** (*p* = 0.54, two-sample *t*-test).

In order to achieve the preferred angle of ~45°, the image acquisition speed should be changed by a factor of *k* = tan(θ). If the line-scan imaging parameters that give 60° streak angle can be changed to improve the acquisition speeds by 30%, i.e., *k* = 1.3, the resultant streaks will have an angle of 53.11° (Equation 2). A streak angle of 45°can be achieved if the same imaging can be made faster by ~73% (*k* = 1.73, Equation 4). The simplest way to change the image acquisition speeds (or Δ*t*, line-scan time) is by changing the spatial resolution, pixel dwell time, and/or decreasing the pixel length of an imaged vessel segment. The caveat here is that the RBC streaks must maintain sufficient contrast to provide a signal-to-noise ratio (SNR) that enables further data processing and velocity measurements on the image (Figure 2).

### Sobel filtering of the line-scan images

Line-scan images often need to be filtered to remove artifacts that create luminance variations unrelated to the flow of RBCs. Artifacts may be static or slowly changing with time (relative to the line-scan duration) such as the artifacts caused by breathing or heartbeats in larger animals. Filtering is commonly performed by temporal demeaning of these space-time images, which is achieved by subtracting the mean value of all the pixels on the time axis at a given point in space. However, temporal demeaning can only address the time-invariant artifacts. Sobel filtering of the line-scan images efficiently removes both types of artifacts.

We used Sobel filtering in the time-dimension of the line-scan images (vertical Sobel filter, since our images have line-scans stacked vertically). The Sobel filter used here is 3 × 3 pixels and is applied by the convolution of the line-scan image *I* (*x*, *t*) with the vertical Sobel operator (*S*) as,

(5) F(x, t)=I(x, t)⊗S

(6) $$S=\left[\begin{array}{ccc} 1 & 2 & 1\\ 0 & 0 & 0\\ -1 & -2 & -1 \end{array}\right]$$

**Figure 3:**
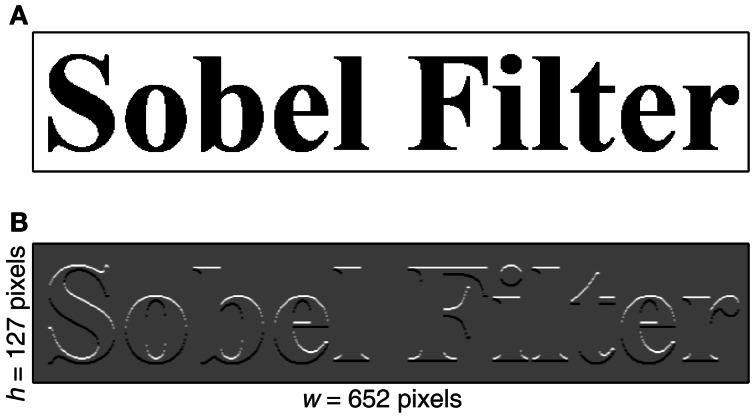
**Effect of Sobel filtering on the image. (A)** An image containing the text “Sobel Filter” is displayed. **(B)** Same image after two-dimensional convolution with a 3 × 3 vertical Sobel operator (Equation 6). Note the enhancement of edge features with horizontal components and disappearance of edge features with vertical components.

Such filtering efficiently enhances horizontal edges and reduces vertical edges (Figure 3). This minimizes slow time-varying components of the line-scan images while sparing the RBC streak edges. We did not find it necessary to use a Sobel operator with a larger kernel size, e.g., 5 × 5 or 7 × 7. Two-photon resolution produces sharp RBC streak edges, the thickness of which does not span more than a pixel or two. Moreover, line-to-line differences in the contrast of streak edges are best enhanced when the kernel height is 3.

### Iterative radon transforms

#### Radon transform

The Radon transform of line-scan images has been described in detail earlier (Drew et al., 2010). Briefly, the Radon transform $\tilde{F}\left(r,\;\theta \right)$ of the image *F* (*x*, *t*) at a given angle θ can be defined as,

(7) $$\begin{array}{l} \tilde{F}\left(r,\;\theta \right)=\int_{-\infty }^{\infty }F\left(x\left(\tau \right),\;t\left(\tau \right)\right)d\tau \\ \;=\int_{-\infty }^{\infty }F\left(\tau \sin(\theta )+r\cos(\theta ),-\tau \cos(\theta )+r\sin(\theta )\right)d\tau \end{array}$$

The angle of the RBC streaks in the line-scan image is determined by the angle θ_max_ at which the maximum variance is found,

(8) $$\theta_{\text{max}}=\underset{\theta \in \left[-\frac{\pi }{2},\;\frac{\pi }{2}\right)}{\text{max}}\text{Var}{\left\{\tilde{F}\left(r,\;\theta \right)\right\}}_{r}$$

Blood velocity (*v*) is then calculated by scaling the tangent of this angle by the temporal (Δ*t*) and spatial (Δ*x*) resolution of the image.

(9) $$v=\frac{\Delta x}{\Delta t}\text{tan}\left(\theta_{\text{max}}\right)$$

**Figure 4:**
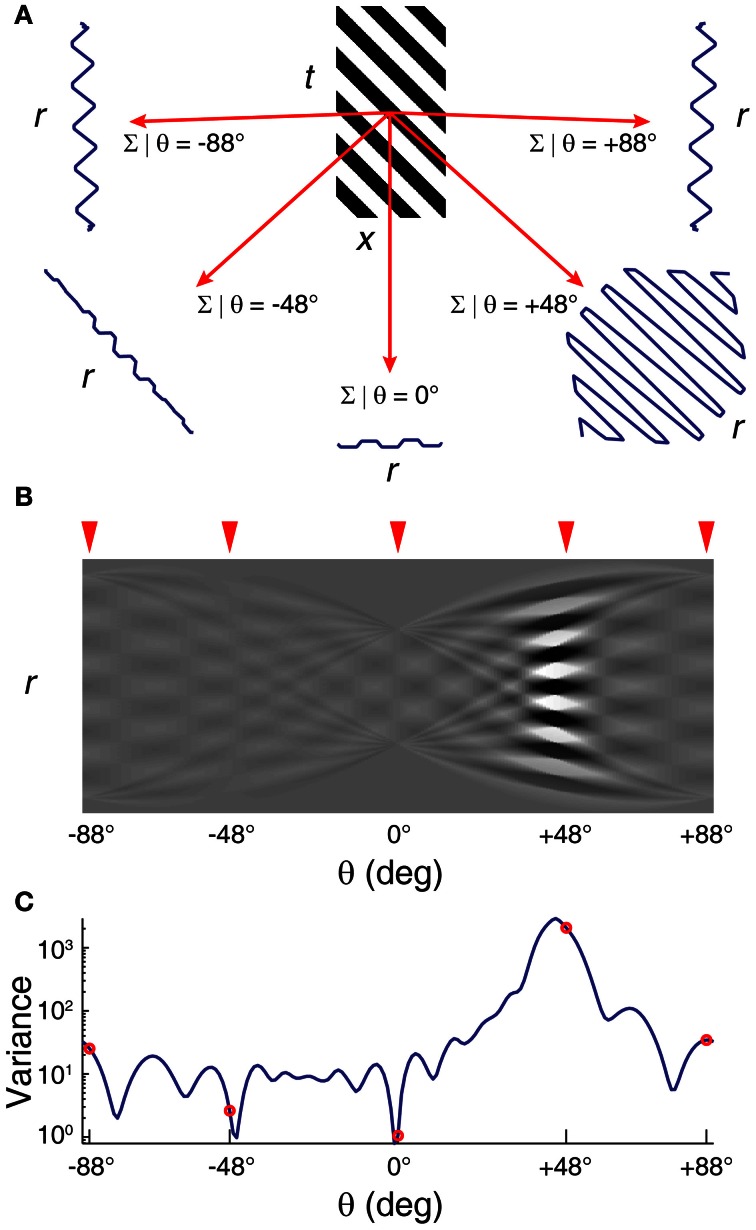
**Radon transform of a striped image *F*(*x,t*) using 1° step-size. (A)** Sample Radon transforms at 5 example angles. **(B)** Radon transform space $\tilde{F}\left(r,\;\theta \right)$ and **(C)** variance at each angle spaced at 1°. Radon transforms at 5 selected angles in **(A)** are indicated by the red arrowheads on the Radon transform space in **(B)** and as red open circles on the variance plot in **(C)**. Peak in the variance at +45° signify the stripe angle of +45°.

One such Radon transform (angles spaced at 1°) is shown in Figure 4.

#### Effect of pixel resolution of the image on the precision of velocity detection

The Radon transform algorithm determines velocity from space-time images by detecting an angle with maximum variance in the Radon transform space of the sampled angle values. While the Radon transform space can be sampled at any set of angles, the angle steps finer than the precision offered by the image only add to the computational load. The angle detection also depends on the size of the space-time image, and on the spatial (Δ*x*) and temporal (Δ*t*) resolution. Therefore, it is important to consider the limitations imposed by these factors on the precision of the angle detection.

A space-time image of width *w* pixels and height *h* pixels (representing the space and time dimensions of the image respectively) would cover the distance *x* and time *t* as follows,

(10) $$\begin{array}{l} x=w\;\cdot \;\Delta x\\ \;t=h\;\cdot \;\Delta t \end{array}$$

**Figure 5:**
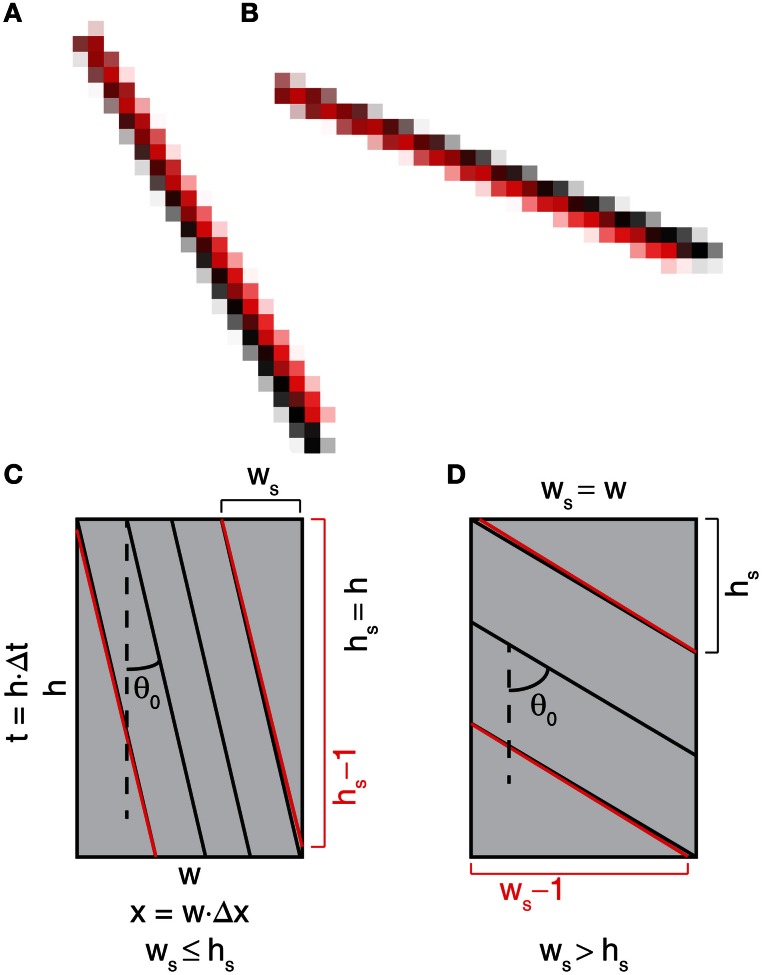
**Detection of the change in the RBC streak angle is constrained by the pixel resolution of the image. (A,B)** Black and red stripes originating from the same pixel but terminating with 2-pixel offset vertically **(A)** or horizontally **(B)**. Noticeable separation of the stripes starts at the mid-point where the stripes are offset by 1 pixel. **(C,D)** Schematic diagram of a space-time image with stripes representing RBC streaks with relatively vertical (|θ_0_| ≤ 45°, **(C)** or horizontal (|θ_0_|> 45°, **(D)** streak angles. Red lines represent change in the RBC streak angle when the end of the streak is offset by 1 pixel in the longer dimension. Also see Materials and Methods for additional details on the detection sensitivity of the streak angle for an image.

The finest detectable change in the angle using the Radon transform for such an image is dependent on the number of streaks (*n*_*s*_) in the image and the finest detectable change in the angle from a single streak (δ_*s*1_). Offsetting a single RBC streak trajectory by one pixel in the longer dimension of the streak can determine δ_*s*1_ (Figure 5). Assuming that only one streak in an image has changed the angle that is detectable with the Radon transform while other streaks maintained the same angle, the theoretically achievable finest detectable change in the angle (δ_*n*_) would be,

(11) $$\delta_{n}=\frac{\delta_{s1}}{n_{s}}$$

In other words, the finest detectable change in the angle improves with an increase in the number of streaks in a given image. For a given RBC streak spanning *w*_*s*_ pixels in the space-dimension and *h*_*s*_ pixels in the time-dimension, δ_*s*1_ and *n*_*s*_ can be calculated as,

(12) $$\delta_{s1}=\{\;\begin{array}{c} \text{arctan}\left(\frac{w_{s}}{h_{s}-1}\right)-\text{arctan}\left(\frac{w_{s}}{h_{s}}\right)\;if\;w_{s}\leq h_{s}\\ \text{arctan}\left(\frac{w_{s}}{h_{s}}\right)-\text{arctan}\left(\frac{w_{s}-1}{h_{s}}\right),\;if\;w_{s}>h_{s} \end{array}$$

(13) $$n_{s}=\left\{ \begin{array}{l} \left\lfloor \frac{x}{d_{s}}\right\rfloor =\left\lfloor \frac{w\;\cdot \;\Delta x}{d_{s}}\right\rfloor \;if\;h_{s}=h\\ \left\lfloor \frac{t}{d_{t}}\right\rfloor =\left\lfloor \frac{h\;\cdot \;\Delta t}{d_{t}}\right\rfloor =\left\lfloor \frac{h\;\cdot \;\Delta x\;\cdot \;w_{s}}{d_{s}\;\cdot \;h_{s}}\right\rfloor \\ \;=\left\lfloor \frac{t\;\cdot \;\Delta x\;\cdot \;w_{s}}{\Delta t\;\cdot \;d_{s}\;\cdot \;h_{s}}\right\rfloor ,\;if\;w_{s}=w \end{array}\right.$$

where *n*_*s*_ is the maximum number of streaks that can fit in the image. *d*_*s*_ and *d*_*t*_ are the minimum possible distance between two streaks in the space (in microns) and time (in seconds) dimensions, respectively. The streak angle (θ_0_) can be calculated by application of the Radon transform on the image. With a known θ_0_, estimation of *w*_*s*_ and *h*_*s*_ for an image of width *w* pixels and height *h* pixels can be made using,

(14) $$\begin{array}{l} w_{s}=\text{min}\left(w,\;\left\lceil h\text{ tan}\left(\theta_{0}\right)\right\rceil \right)\\ \;h_{s}=\text{min}\left(h,\;\left\lceil w\text{ cot}\left(\theta_{0}\right)\right\rceil \right) \end{array}$$

The precision of angle detection is dependent on image dimensions (*w* and *h*), the detected RBC streak angle from the image (θ_0_) and the number of RBC streaks that can fit in a recorded line-scan (*n*_*s*_, which is in turn dependent on Δ*x*, *w*, and *d*_*s*_). The actual distance between RBC streaks can be greater than *d*_*s*_, making the actual number of streaks less than *n*_*s*_. Therefore, the realistic angle precision from a given image can be lower than δ_*n*_ but higher than δ_*s*1_.

#### Detection of change in the velocity is dependent on streak angle

Change in the blood velocity in a given vessel depends on a number of factors such as vessel size, vessel type (artery, capillary, vein), anatomical location, and stimulus type and can range from 0 to 80% of the baseline blood velocity (Drew et al., 2011; Shen et al., 2012). Therefore, the sensitivity of the algorithm needs to accommodate the expected change in the blood velocity without being computationally expensive. This can be achieved by optimizing the angle step-size used for the Radon transform in order to detect the desirable change in velocity.

The fractional change in velocity (Δ*v*/*v*) can be defined as,

(15) $$\frac{\Delta v}{v}=\frac{v_{1}-v_{0}}{v_{0}}=\frac{v_{1}}{v_{0}}-1$$

Where *v*_1_ is new velocity and *v*_0_ is initial velocity. The corresponding change in RBC streak angle, δ = |θ_1_ − θ_0_|, can be computed by combining Equations 9 and 15 and rearranging the terms,

(16) $$\frac{\Delta v}{v}=\frac{\text{tan}(\theta_{\text{1}})}{\text{tan}(\theta_{\text{0}})}-1=\frac{\text{tan}\left(\theta_{0}\pm \delta \right)}{\text{tan}(\theta_{\text{0}})}-1$$

(17) $$\delta =\left|\theta_{1}-\theta_{0}\right|=\left|\text{arctan}\left(\left(\frac{\Delta v}{v}+1\right)\text{tan}(\theta_{\text{0}})\right)-\theta_{0}\right|$$

**Figure 6:**
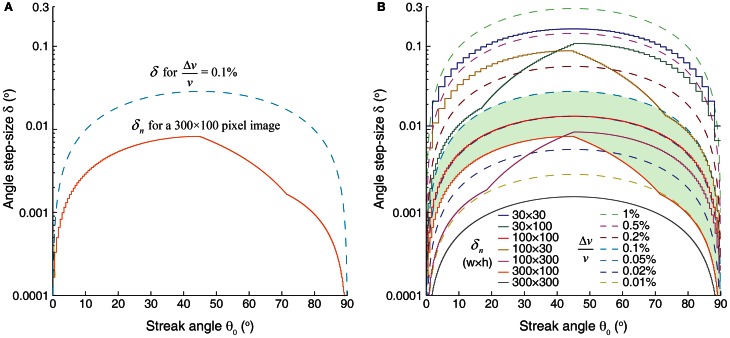
**Detection of fractional change in the velocity is dependent on the RBC streak angle and image dimensions. (A)** Range of angle step-sizes (δ) that can be used in order to detect fractional change in the velocity (Δ*v*/*v*) of 0.1% on a 300 × 100 pixel image. Upper limit of δ that can be used is delineated by the blue dashed line. The solid orange line demarcates the detection sensitivities of -change in the angle (δ_*n*_) that can be resolved on image dimensions of 300 × 100 pixels for given streak angles. δ-values between these two curved lines should be used in order detect Δ*v*/*v* of 0.1%. **(B)** Limits imposed on δ by the required Δ*v*/*v* (dashed lines) and δ_*n*_ for different image sizes (solid lines). Green shaded area corresponds to the δ values as shown in **(A)**. In order to achieve a given Δ*v*/*v* sensitivity, δ values should be below the respective Δ*v*/*v* curve, but above the δ_*n*_ curve as determined by the image pixel dimensions. Note that the tolerance in the angle step-size values is highest at 45° streak angle. For a given image size an angle step-size value below the solid line is not resolvable. δ_*n*_ calculations are based on a space-time image with 1.19 μm/pixel spatial resolution and inter-streak distance of 4 μm (~3.3 pixels).

Thus, for the given RBC streak angle (θ_0_), δ can be determined instantaneously because it is dependent on the desired Δ*v*/*v*. Likewise, the precision of Δ*v*/*v* can be calculated from the value of δ used in the Radon transform and θ_0_. Note that the value of δ that can provide a given Δ*v*/*v* is the highest for θ_0_ = 45°. Therefore, it is advantageous to perform line-scan imaging that yields ~45° streak angles. The smallest detectable δ, and therefore Δ*v*/*v*, is dependent on the pixel dimensions of the image and θ_0_. δ smaller than the theoretically achievable finest detectable change in the angle (δ_*n*_) cannot be resolved (see Equations 11–14 and Figure 6).

#### Application of the iterative radon transform algorithm

The size of angle steps used in the calculation of Radon transforms determines the precision of angle measurements. This creates a trade-off between the achieved precision of angle measurements and the computational load. In the first implementation of the Radon transform to calculate streak angle (Drew et al., 2010), this trade-off was addressed by a two-step adaptive algorithm. First, relatively coarse angle steps of 1° were used to achieve the approximate angle. Second, finer angle steps of 0.25° around the measured angle were used. We refine the application of Radon transforms in a multi-step iterative manner to further optimize the balance between computational speed and precision of angle measurements.

**Figure 7:**
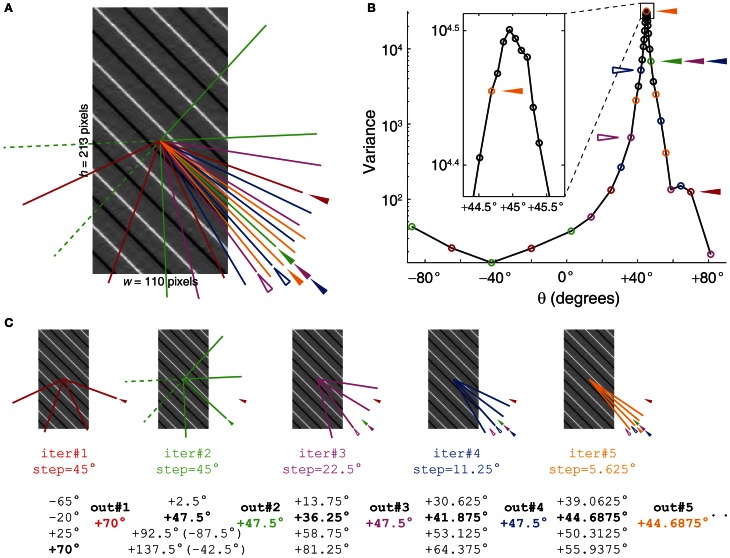
**Iterative Radon transform with progressively finer angle steps on subsequent iterations leads to precise angle measurements. (A)** An image with 45° stripes after Sobel filtering. Color-matched lines represent angles of the first five Radon transform iterations. Additional iterations continue with step-size and angular span values that are half of the preceding iteration. **(B)** Radon variance plot, showing the variance of the Radon transform at 40 different angles corresponding to 10 iterations. The final angle step-size (δ) was approximately 0.0879° (Equation 19), sufficient to detect ~0.3% fractional change in the velocity (Δ*v*/*v*) for this particular streak angle (45°, Equation 16). The variance values are represented with open circles and the first five iterations are color-matched with the lines shown in **(A)**. Filled arrowheads of the corresponding colors in **(A)** and **(B)** represent the angle values for given iterations that were carried forward to the next iteration. Open arrowheads represent the angle values with the maximum variance for given iterations, but smaller than the one from the previous iterations, and therefore were not carried forward to the next iteration. **(C)** Algorithm outputs for the first five iterations showing the angle range, angle step-size, and final angle output at the end of the iteration. The angles with the maximum variance for a given iteration are marked in bold. Angle value 47.5° was found to have maximum variance in iteration 2, and was carried forward in proceeding iterations 3 and 4. This was because the maximum variances for the angle values in those iterations were smaller than that for 47.5° (Compare variances represented by open and filled arrowheads in **(B)**. In this example, the iterative Radon transform offered a 12-fold increase in the angle precision and was still 4.5-times faster than 180 successive Radon transforms with angles spaced by 1° (see Materials and Methods).

The iterative Radon transform is started by first applying Radon transforms in sparse angle space and then finding the angle that gives the maximum variance in the image. In subsequent iterations, sets of angles are used that are less sparse than the preceding iteration and centered on the angle that yielded the maximum variance in the previous iterations (Figure 7). The smallest angle step-size (δ) to be used for a given image depends on the δ_*n*_, θ_0_, and the desired Δ*v*/*v* (Equations 11, 17). Since the variance at 0° (vertical) is 0 when using a vertical Sobel filter, an angle selection of 0° should be avoided.

The first iteration of the algorithm uses four Radon transforms with a 45° angle step-size in order to span the full range of 180°. In the second iteration, again four Radon transforms with a 45° angle step-size are used with the center on the angle that yielded the maximum variance in the first iteration. Thus, the first two iterations give an angle resolution of 22.5°. In the subsequent iterations, four Radon transforms with an angle step-size half that of the previous iteration are used. i.e., 22.5° in the third iteration, 11.25° in the fourth iteration, and so on. The number of iterations, *i*_irt_, required for achieving a given angle precision can be described as,

(18) $$i_{\text{irt}}=\left\lceil {\text{log}}_{2}\left(\frac{45}{\delta }\right)\right\rceil +1$$

Conversely, the precision achieved by a given number of iterations would be,

(19) $$\delta =\frac{45}{2^{i_{\text{irt}}-1}}$$

Since each iteration performs four Radon transforms, the total number of Radon transforms (*n*_irt_) used in the iterative Radon transform algorithm is,

(20) $$n_{\text{irt}}=4i_{\text{irt}}$$

## Results

### Effects of sobel filtering on measured streak angle accuracy

**Figure 8:**
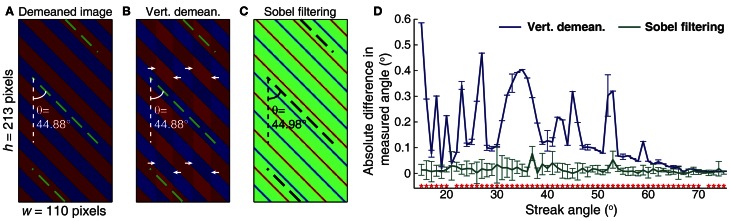
**Effect of Sobel filtering on the accuracy of angle measurements when using the Radon transform. (A)** Radon transforms on a simulated image of 45° orderly streaks after subtracting the mean luminance from the image. Resulting angle of 44.88° (dashed green lines) represents a 0.12° deviation from the actual angle. **(B)** Vertical demeaning leads to the same angle measurement as shown in **(A)**. The orderly vertical bands (highlighted by white arrows) are the result of different mean luminance across columns of the original image. **(C)** Vertical Sobel filtering of the image shown in **(A)** gives 44.98° (dashed black lines) on the Radon transform, an error of 0.02°. Thirteen iterations of the Radon transforms (52 Radon transforms) resulted in an angle step-size of ~0.002° (see Equation 19). **(D)** Sobel filtered images offer more accurate angle measurements when compared with vertically demeaned images. Mean ± SD of absolute difference in measured angles on simulated images are shown. Each angle value consists of results from *n* = 36 images with streaks at different spatial locations. Statistically significant differences are represented by a red asterisk for a given angle (*p* < 0.05, two-sample *t*-test).

Since for blood velocity the feature of interest in a given line-scan image is the streaks resulting from plasma-RBC “junctions,” it is important to ignore all the other elements of the image by minimizing them and/or enhancing the plasma-RBC junction. The vertical Sobel filter achieves this by removing low frequency changes in pixel luminance in the time dimension of the image. Removing the aggregate mean of the image by subtracting it from each pixel of the image (whole-image demeaning) and removing the mean value across time at each spatial point of the line (temporal demeaning, vertical in our case) has been proposed previously for pre-processing of the image before performing Radon transforms (Drew et al., 2010). However, these methods do not effectively minimize the column-to-column variations in the luminance. Such differences are more pronounced when the image segments used for the Radon transform are small and/or the widths of the RBC streaks are wide for the given dimensions of the image segments (Figure 8A). Although subtle, these column-to-column variations in luminance (Figure 8B) lead to sub-optimal detection of streak angles. Application of Sobel filtering minimizes these variations and detected angles become more accurate (Figure 8C). For instance, the Radon transform of an image with 45° streak angle gives an angle of 44.88° after vertical demeaning while the same image after Sobel filtering gives an angle of 44.98°. This error of 0.1° at a 45° streak angle can lead to an error of ~0.35% in detected Δ*v*/*v* (Equation 16). To further confirm the accuracy offered by Sobel filtering, we compared the differences in the measured angles from the actual angles on simulated line-scan images that were Sobel filtered versus vertically demeaned. A range of angles was tested with each angle having a sample size of *n* = 36 images (112 × 215 pixels, *w* × *h*), differing slightly in the spatial position of the streak. We found that angles measured on Sobel filtered images are more accurate when compared with the same image sequences that were vertically demeaned (Figure 8D). For much longer time-dimension images (200 × 1000 pixels, *w* × *h*), the improved accuracy on Sobel filtered images was retained, even though overall error rates were reduced with both types of filtering (data not shown).

**Figure 9:**
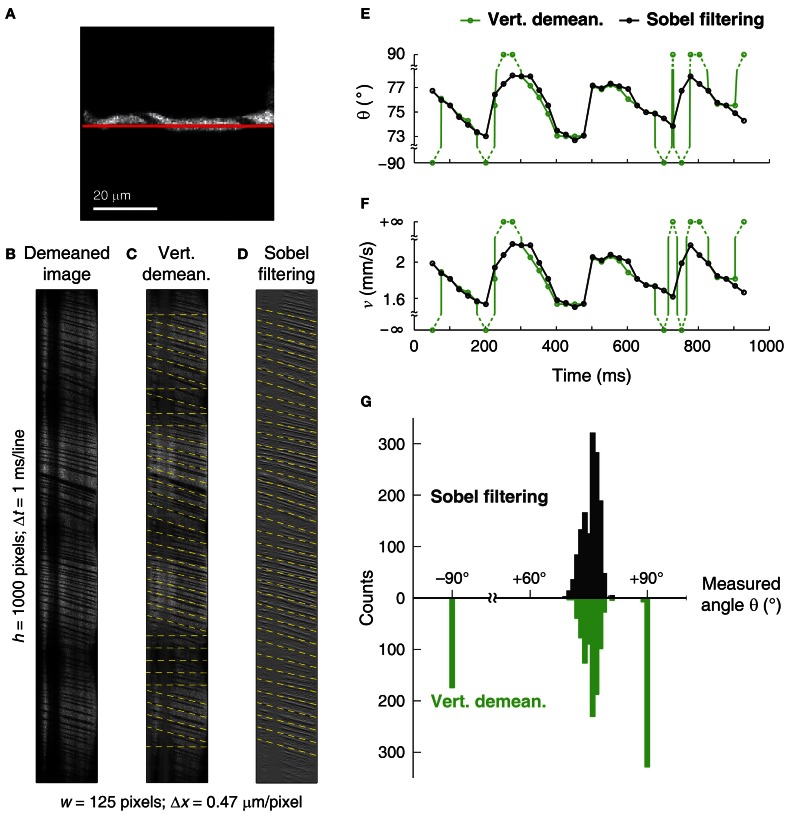
**Improvement in the angle measurement using Sobel filtering on an image with motion artifacts. (A)** Reference full-frame image of a 5 μm diameter microvessel in the cat visual cortex. The line-scan trajectory is shown as a red line. **(B)** Line-scan image from the microvessel. Continuous dark vertical bands are due to line-scan path going out of the vessel and the intermittent horizontal dark bands are related to brain motion due to transmitted heartbeat movements. **(C)** Vertically (temporally) demeaned image of **(B)** leads to incorrect angle and velocity estimation at some image segments (see horizontal dashed yellow lines). Note the suppression of time-invariant vertical bands, but virtually unchanged time-varying horizontal bands. **(D)** Sobel filtered image of **(B)** leads to suppression of both types of artifacts and enhancement of RBC streak edges. Dashed yellow lines represent measured angles over image segments of 100 lines. **(E,F)** Angle **(E)** and velocity **(F)** measurements obtained after vertical demeaning (green traces) and Sobel filtering (black traces). Circles represent the dashed lines shown in **(C)** and **(D)**. Incorrectly measured angle/velocity values are connected with dashed lines and correspond to horizontal dashed lines in **(C)**. **(G)** Distribution of measured angles after Vertical demeaning (green) or Sobel filtering (black). A total of 35,000 contiguous line-scans were collected from the microvessel shown in **(A)** to create 1396 overlapping 100-line image segments. Incorrectly measured angles are clustered at ±90° and evident only after vertical demeaning. δ of ~0.011° was used in order to achieve Δ*v*/*v* of <0.1% (see Equation 17) on the overlapping image segments of 100 lines which offered δ_*n*_ of ~0.003° (see Equations 11–14).

**Figure 10:**
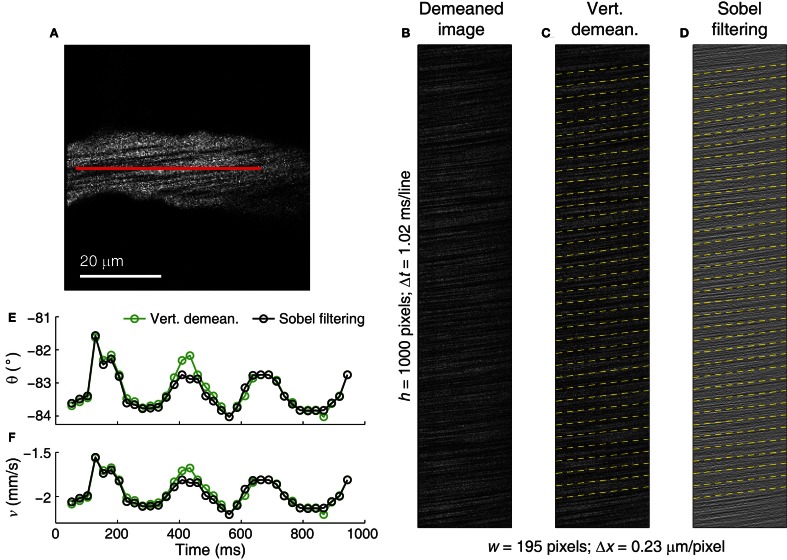
**Application of Sobel filtering on an image with thin RBC streaks. (A)** Reference full-frame image of a 14 μm diameter venule in the cat visual cortex. The line-scan trajectory is shown as a red line. **(B)** Line-scan image from the microvessel showing thin, near-horizontal RBC streaks. **(C)** Vertically demeaned image of **(B)** showing measured RBC streak angle and velocity (dashed yellow lines). **(D)** Sobel filtered image of **(B)** shows RBC streak enhancement, measured RBC streak angle and velocity. Dashed yellow lines represent measured angles over image segments of 100 lines. **(E,F)** Angle **(E)** and velocity **(F)** traces after vertical demeaning (green traces) and Sobel filtering (black traces). Circles represent the dashed lines shown in **(C)** and **(D)**. Subtle difference in the values is the result of different filtering (also see Figure 8). δ of ~0.0055° was used in order to achieve Δ*v*/*v* of <0.1% (see Equation 17) on the overlapping image segments of 100 lines which offered δ_*n*_ of <0.001° (see Equations 11–14).

In line-scan images acquired for blood velocity measurements, the Sobel filter acts as a high-pass filter by enhancing RBC streak margins while suppressing small line-to-line changes in pixel luminance. As a result, only the angle of RBC streak margins (plasma-RBC junctions) provides high variance in the Radon transform space. This improves the accuracy and precision of measuring the angle, and in turn velocity. Sobel filtering can be applied to line-scan images which have both thick (Figure 9) and thin (Figure 10) RBC streaks. In line-scan images contaminated by brain movements that are due to transmitted pulsations from heartbeats (Figure 9B), vertical demeaning fails to suppress time-varying artifacts (Figure 9C) and leads to incorrect RBC streak angle detection (Figure 9E). Apart from suppressing artifacts, Sobel filtering also enhances the RBC streak edges (Figure 9D) leading to an angle measurement with the iterative Radon transform algorithm that matches the actual RBC streak angle and therefore velocity (Figure 9F). We also compared the angles measured after Sobel filtering vs. vertical demeaning over a long sequence of 35,000 contiguous line-scans (see Figure 9G). We found that ~37% (*n* = 515) of image segments after vertical demeaning failed to accurately measure RBC streak angles, but no such errors were obtained when using Sobel filtered image segments (Figure 9G).

Sobel filtering preserves the streak angle information in the images with thin, relatively horizontal RBC streaks (Figure 10). In line-scan images with very thin RBC streaks (Figure 10B), the Sobel filter enhances the RBC streak edges (Figure 10D) when compared with vertical demeaning (Figure 10C), leading to detected angle values that are different from the vertically demeaned image (Figure 10E). The ~0.6° difference in the computed angle between the two filtering methods at ~0.45 s in Figure 10E at the RBC streak angle of ~83° translates into a Δ*v*/*v* of ~8% (Equation 16), or ~0.16 mm/s in this case (Figure 10F). As the streaks become more horizontal, the difference between true and measured angle becomes similar after both vertical demeaning and Sobel filtering (see Figure 8D). Therefore, in the absence of time-varying artifacts, Sobel filtering provides little enhancement for images with very horizontal streaks.

### Improved speed and precision of velocity measurements with iterative radon transforms

Blood velocity measurements can only be as precise as the angle step-size used in the Radon transform. But decreasing the step-size to improve precision results in additional Radon transforms and increased computation time. The iterative Radon transform method helps overcome this trade-off. The total number of Radon transforms required by the traditional non-iterative method (*n*_trt_) spanning the full range of 180° for the step-size (δ) would be,

(21) $$n_{\text{trt}}=\left\lceil \frac{180}{\delta }\right\rceil$$

Compared to the traditional non-iterative method, the method described here requires an order of magnitude fewer Radon transforms. Based on Equations 18, 20, and 21, the relationship between the total number of Radon transforms required by the traditional non-iterative method (*n*_trt_) and the iterative method (*n*_irt_) is,

(22) $$n_{\text{irt}}=4\left\lceil {\text{log}}_{2}\left(\frac{n_{\text{trt}}}{2}\right)\right\rceil$$

**Figure 11:**
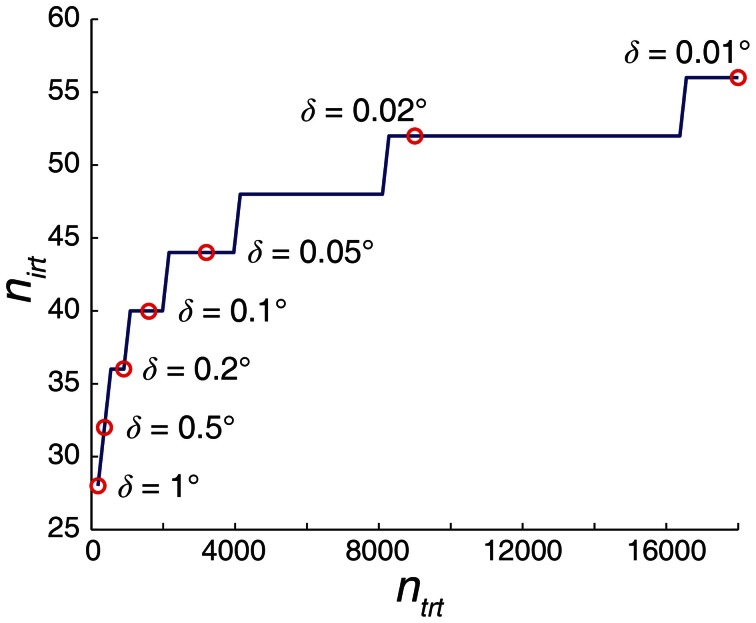
**Iterative Radon transform method markedly reduces the required number of Radon transforms (*n*_irt_) to achieve a given angle precision (δ) when compared with non-iterative method (*n*_trt_).** The number of Radon transforms in the iterative algorithm increases logarithmically as δ becomes finer, compared to a linear increase for the traditional, non-iterative counterpart. As a result, there is an exponential increase in the computation speed for the iterative algorithm compared to the traditional non-iterative Radon transform methods. Results are based on Equations 18–22.

The angle precision (δ) of 1° requires 180 Radon transforms with the non-iterative method but only 28 using the iterative method. Similarly, an angle precision of 0.01° would require 18,000 Radon transforms if performed non-iteratively, but can be resolved with only 56 Radon transforms using the iterative method (Figure 11). The iterative Radon transform method overcomes the speed-precision trade-off by starting with sparse sampling of the angles and then sampling progressively finer angles in the subsequent iterations centered around the angle with the highest variance (see Materials and Methods).

## Discussion

The hybrid algorithm presented here performs pre-processing of the image followed by iterative application of Radon transforms. Pre-processing of the images with Sobel filtering has an advantage over vertical demeaning in that it removes the slow time-varying artifacts in addition to time-invariant artifacts. This is especially useful in large animal preparations where brain movement artifacts due to respiration and heartbeats are sometimes difficult to eliminate. Such artifacts lead to line-scan images with discontinuous RBC streaks and/or uneven or varying background luminance. Additionally, Sobel filtering also enhances the RBC streak margins. Other types of filtering using operators similar to Sobel, e.g., Prewitt or Scharr operators can also be used with equivalent results.

Sobel filtering results in an increased contrast at the plasma-RBC junctions. Because the Sobel operator we use is oriented vertically, the margins of relatively horizontal streaks are enhanced more than vertical streaks. Completely vertical RBC streaks appear in cases of stagnant blood flow (Kleinfeld et al., 1998). In these situations, any vertical operator (including Sobel, vertical demeaning) will suppress the streaks and therefore should be avoided. The detection of the correct angle using the iterative Radon transform is dependent on the SNR of the Sobel filtered image. We found in our simulations that almost vertical stripes (angle < 1°) can be efficiently detected using the iterative Radon transform algorithm after Sobel filtering. However, the detection is less robust to noise than in the case of stripes with higher angle values. More generally, velocity measurements using the algorithm described here will produce the best results provided that the angles of the RBC streaks are close to 45° (see Materials and Methods).

Changes in velocity due to heart rate pulsations can be substantial (see Figures 9, 10) and may exceed the changes due to sensory stimulus-evoked responses. However, the Δ*v*/*v* induced by heart rate pulsations has the frequency range of 3–8 Hz which is much higher than a typical <1 Hz Δ*v*/*v* evoked by a given sensory stimulus (Kleinfeld et al., 1998; Chaigneau et al., 2003; Berwick et al., 2008; Drew et al., 2011; Shen et al., 2012). Therefore, such physiological Δ*v*/*v* can be band-pass filtered using Fourier transform methods to generate more accurate measurements of stimulus-evoked Δ*v*/*v*. A low-pass filtering can also be achieved by averaging the velocities over time-windows that span multiple cardiac cycles. Alternatively, the collected line-scan data can be consistently phase-locked to the cardiac cycle by triggering line-scan imaging at a particular phase of the cardiac cycle, e.g., using QRS complex as a trigger. The angle step-size (δ) we used for Figures 9, 10 were ~0.01° and ~0.005°, respectively, in order to achieve the desired detection sensitivities of 0.1% Δ*v*/*v* (see Equation 17). However, if the experimental paradigm does not demand detection of minute changes and/or wider range in the velocities, the user may choose a bigger δ and/or narrow starting angle range for even faster computation speeds.

Iterative application of the Radon transform resulted in an order of magnitude faster determination of blood velocity when compared with other algorithms of blood velocity measurements. When testing simulated line-scan data with RBC streak shifts ranging from 10 to 80 pixels per line and using ~0.1° angle precisions on a 200 × 15,000 image, our algorithm was ~6.5× faster than the correlation-based method (Kim et al., 2012). However, the correlation-based method offers superior velocity measurements in some instances of near-horizontal RBC streaks. A spatial frequency-based analysis claims 1.25× faster performance over Radon transform when using 1° angle step-size spanning 180° (Autio et al., 2011). Our algorithm achieves this precision in only 28 Radon transforms (Equation 22), making it 6.4× faster than the Radon transform when applied in a non-iterative manner. Thus, the advantage offered by the iterative implementation makes our algorithm 5.14× faster compared with a spatial frequency-based analysis. The “fasttrack” algorithm that uses global energy minimization technique claims superior velocity measurements than Radon transform in near-horizontal RBC streaks, but is generally 5× slower than traditional Radon transform (Deneux et al., 2011, 2012). Conservatively estimating a 6.4× speed improvement offered by the iterative implementation of Radon transforms over its non-iterative counterpart makes our algorithm 32× faster than the “fasttrack” algorithm. Furthermore, Sobel filtering followed by very fine angle measurements in an iterative manner eliminates the likelihood of detecting false angles that can otherwise occur when line-to-line discontinuities of RBCs are present in space-time images. The global energy minimization technique appears to have a comparable accuracy to the standard Radon transform method when RBC streaks are not near horizontal (Deneux et al., 2011). The apparently poor performance of the Radon transform algorithm on detecting near-horizontal RBC streaks (high velocities) in the study by Deneux and colleagues may be due the use of relatively coarse angle steps and incomplete removal of slow time-varying luminance variations in the images that are unrelated to the flow of RBCs.

In summary, we present a method of accurately and precisely measuring blood velocities from line-scan images in a computationally efficient manner. First, Sobel filtering is used to remove slow time-varying artifacts and enhance the edges of RBC streaks. This leads to accurate estimation of RBC streak angles and thus blood velocity measurements. Second, the iterative Radon transform minimizes the required number of Radon transforms needed to precisely estimate the RBC streak angle. This leads to fast RBC streak angle measurements. The near-ideal precision attained with the iterative Radon method together with the high accuracy provided by Sobel filtering ensures that relatively small differences in baseline vs. stimulus-evoked RBC streak angles can be measured. This is a non-trivial advance because data collected from *in vivo* experiments include movement artifacts. Such data may otherwise be discarded, or if used would lead to erroneous measurements. More generally, the individual components of our algorithm can be used independently for a broad range of particle velocity applications. For example, Sobel filtering of noisy space-time images collected for tracking the movement of any particle can lead to improvement in the SNR by enhancing the particle streak edges. The concept of iterative image analysis can be used to speed-up other algorithms that have been used to calculate the slope of RBC streaks (Drew et al., 2010; Duncan et al., 2010, Autio et al., 2011). Our hybrid algorithm (in its entirety or as individual components) can also be used for real-time implementation of velocity calculations in high-speed imaging applications that use resonant or acousto-optic deflector scanners.

### Conflict of interest statement

The authors declare that the research was conducted in the absence of any commercial or financial relationships that could be construed as a potential conflict of interest.

## Supplementary material

The source code to run the hybrid algorithm is available online at: http://www.frontiersin.org/Brain_Imaging_Methods/10.3389/fnins.2013.00106/abstract and Matlab Central File Exchange at: http://www.mathworks.com/matlabcentral/fileexchange/42019-hybridvel

## References

[B1] Akerboom J. Carreras Calderon N. Tian L. Wabnig S. Prigge M. Tolo J. (2013). Genetically encoded calcium indicators for multi-color neural activity imaging and combination with optogenetics. Front. Mol. Neurosci. 6:2 10.3389/fnmol.2013.00002 23459413 PMC3586699

[B2] Autio J. Kawaguchi H. Saito S. Aoki I. Obata T. Masamoto K. (2011). Spatial frequency-based analysis of mean red blood cell speed in single microvessels: investigation of microvascular perfusion in rat cerebral cortex. PLoS ONE 6:e24056 10.1371/journal.pone.0024056 21887370 PMC3161111

[B3] Berwick J. Johnston D. Jones M. Martindale J. Martin C. Kennerley A. J. (2008). Fine detail of neurovascular coupling revealed by spatiotemporal analysis of the hemodynamic response to single whisker stimulation in rat barrel cortex. J. Neurophysiol. 99, 787–798 10.1152/jn.00658.2007 18046008 PMC2652198

[B4] Chaigneau E. Oheim M. Audinat E. Charpak S. (2003). Two-photon imaging of capillary blood flow in olfactory bulb glomeruli. Proc. Natl. Acad. Sci. U.S.A. 100, 13081–13086 10.1073/pnas.2133652100 14569029 PMC240748

[B5] Deneux T. Faugeras O. Takerkart S. Masson G. S. Vanzetta I. (2011). A new variational method for erythrocyte velocity estimation in wide-field imaging *in vivo*. IEEE Trans. Med. Imaging 30, 1527–1545 10.1109/TMI.2011.2131151 21427018

[B6] Deneux T. Takerkart S. Grinvald A. Masson G. S. Vanzetta I. (2012). A processing work-flow for measuring erythrocytes velocity in extended vascular networks from wide field high-resolution optical imaging data. Neuroimage 59, 2569–2588 10.1016/j.neuroimage.2011.08.081 21925275

[B7] Drew P. J. Blinder P. Cauwenberghs G. Shih A. Y. Kleinfeld D. (2010). Rapid determination of particle velocity from space-time images using the Radon transform. J. Comput. Neurosci. 29, 5–11 10.1007/s10827-009-0159-1 19459038 PMC4962871

[B8] Drew P. J. Shih A. Y. Kleinfeld D. (2011). Fluctuating and sensory-induced vasodynamics in rodent cortex extend arteriole capacity. Proc. Natl. Acad. Sci. U.S.A. 108, 8473–8478 10.1073/pnas.1100428108 21536897 PMC3100929

[B9] Duncan D. D. Lemaillet P. Ibrahim M. Nguyen Q. D. Hiller M. Ramella-Roman J. (2010). Absolute blood velocity measured with a modified fundus camera. J. Biomed. Opt. 15:056014 10.1117/1.3494565 21054108 PMC2966492

[B10] Kamoun W. S. Chae S. S. Lacorre D. A. Tyrrell J. A. Mitre M. Gillissen M. A. (2010). Simultaneous measurement of RBC velocity, flux, hematocrit and shear rate in vascular networks. Nat. Methods 7, 655–660 10.1038/nmeth.1475 20581828 PMC2921873

[B11] Kim T. N. Goodwill P. W. Chen Y. Conolly S. M. Schaffer C. B. Liepmann D. (2012). Line-scanning particle image velocimetry: an optical approach for quantifying a wide range of blood flow speeds in live animals. PLoS ONE 7:e38590 10.1371/journal.pone.0038590 22761686 PMC3383695

[B12] Kleinfeld D. Mitra P. P. Helmchen F. Denk W. (1998). Fluctuations and stimulus-induced changes in blood flow observed in individual capillaries in layers 2 through 4 of rat neocortex. Proc. Natl. Acad. Sci. U.S.A. 95, 15741–15746 10.1073/pnas.95.26.15741 9861040 PMC28114

[B13] Levy M. Schramm A. E. Kara P. (2012). Strategies for mapping synaptic inputs on dendrites *in vivo* by combining two-photon microscopy, sharp intracellular recording, and pharmacology. Front. Neural Circuits 6:101 10.3389/fncir.2012.00101 23248588 PMC3521157

[B14] O'Herron P. Shen Z. Lu Z. Schramm A. E. Levy M. Kara P. (2012). Targeted labeling of neurons in a specific functional micro-domain of the neocortex by combining intrinsic signal and two-photon imaging. J. Vis. Exp. 70:e50025 10.3791/50025 23271035 PMC3567167

[B15] Ohki K. Chung S. Ch'ng Y. H. Kara P. Reid R. C. (2005). Functional imaging with cellular resolution reveals precise micro-architecture in visual cortex. Nature 433, 597–603 10.1038/nature03274 15660108

[B16] Schaffer C. B. Friedman B. Nishimura N. Schroeder L. F. Tsai P. S. Ebner F. F. (2006). Two-photon imaging of cortical surface microvessels reveals a robust redistribution in blood flow after vascular occlusion. PLoS Biol. 4:e22 10.1371/journal.pbio.0040022 16379497 PMC1324794

[B17] Shen Z. Lu Z. Chhatbar P. Y. O'Herron P. Kara P. (2012). An artery-specific fluorescent dye for studying neurovascular coupling. Nat. Methods 9, 273–276 10.1038/nmeth.1857 22266543 PMC3392962

[B18] Smith K. (2012). Brain imaging: fMRI 2.0. Nature 484, 24–26 10.1038/484024a 22481337

[B19] Sobel I. (1978). Neighborhood coding of binary images for fast contour following and general binary array processing. Comput. Graph. Image Process. 8, 127–135 10.1016/S0146-664X(78)80020-3

[B20] Stosiek C. Garaschuk O. Holthoff K. Konnerth A. (2003). *In vivo* two-photon calcium imaging of neuronal networks. Proc. Natl. Acad. Sci. U.S.A. 100, 7319–7324 10.1073/pnas.1232232100 12777621 PMC165873

[B21] Tian L. Hires S. A. Mao T. Huber D. Chiappe M. E. Chalasani S. H. (2009). Imaging neural activity in worms, flies and mice with improved GCaMP calcium indicators. Nat. Methods 6, 875–881 10.1038/nmeth.1398 19898485 PMC2858873

[B22] Tsien R. Y. (1988). Fluorescence measurement and photochemical manipulation of cytosolic free calcium. Trends Neurosci. 11, 419–424 10.1016/0166-2236(88)90192-0 2469158

[B23] Zhang S. Boyd J. Delaney K. Murphy T. H. (2005). Rapid reversible changes in dendritic spine structure *in vivo* gated by the degree of ischemia. J. Neurosci. 25, 5333–5338 10.1523/JNEUROSCI.1085-05.2005 15930381 PMC6724996

