# Supplementary material for: Improved blood velocity measurements with a hybrid image filtering and iterative Radon transform algorithm
Source: Front Neurosci. 2013 Jun 18;7:106. doi: 10.3389/fnins.2013.00106 (PMC3684769; doi:10.3389/fnins.2013.00106)
Supplement: Supplementary file 1 [file DataSheet1.ZIP › hybridvelexec.html]

hybridvelexec


 


## Contents

- Initialize
- Process the image with different filters
- Iterative Radon transform

```
% [angle,utheta,uvar] = hybridvel(imread('fig9im.tif'),1,[],0.47,1,100,25,[1 125]);
```

## Initialize

```
% load the image
imgtitle = 'fig9im.tif';
inputimg = imread(imgtitle);

% set up parameters
imsize = size(inputimg);
inputimg = double(inputimg);
anglineth = 3;
dvov = 0.1/100; % dv/v to determine minimum step-size
firstthetastep = 45; thetarange = [0 179];
ds = 4; % 4 um streak distance
showimg = 1;
delx = 0.47; % microns/pixel
delt = 1; % ms/line
hi = 100;
lineskip = 25;
xrange = [1 125];

if ~isempty(xrange) && length(xrange)<3
    if length(xrange)==1
        wi = xrange; xrange = [1 wi];
    else
        if xrange(1)>imsize(2)-1
            xrange(1)=1;
        end
        if xrange(2)>imsize(2)-1
            xrange(2)=imsize(2)-2;
        end
        if xrange(2)<xrange(1)
            xrange = [xrange(1) xrange(2)];
        end
        wi = xrange(2)-xrange(1)+1;
    end
else
    wi = imsize(2)-2; xrange = [1 wi];
end

warning off
```

## Process the image with different filters

```
curimtitle = {'edm','vdm','sob'}; curcolor = {'b','g','k','r','c','m'};
imgseg(:,:,1) = inputimg(2:end-1,2:end-1)-mean(mean(inputimg(2:end-1,2:end-1))); % element-wise demean
imgseg(:,:,2) = bsxfun(@minus,inputimg(2:end-1,2:end-1),...
    mean(inputimg(2:end-1,2:end-1),1)); % vertical demean
imgseg(:,:,3) = filter2([1 2 1; 0 0 0; -1 -2 -1],inputimg,'valid');  % 3x3 vertical Sobel filter, Eq. 5,6

imgsegsz = size(imgseg);
firstiter = (thetarange(1):firstthetastep:thetarange(2));
firstiter = firstiter-(firstiter(end)-firstiter(1))/2+1;

segend = hi:lineskip:imgsegsz(1);
segstart = segend-hi+1;
segn = length(segstart);

angle = nan(segn,9,imgsegsz(3)); % angle = [angle,minstep,loc(pix),dels1,deln,%dv/v,iter,irl,speed(mm/s)]
utheta = cell(segn,imgsegsz(3));
uvar = cell(segn,imgsegsz(3));
```

## Iterative Radon transform

```
for ii = 1:imgsegsz(3)
    for jj = 1:segn
        irl = 1; % iterative radon level
        curangle = 0; alltheta = []; allvar = []; iter = 0; thetastep = firstthetastep; curvarmax = 0;
        curimgseg = imgseg(segstart(jj):segend(jj),xrange(1):xrange(2),ii);
        if ii==1
            curimgseg = curimgseg-mean(curimgseg(:)); % element-wise demean
        end
        if ii==2
            curimgseg = bsxfun(@minus,curimgseg,mean(curimgseg,1)); % vertical demean
        end
       while irl
            iter = iter+1;
            % smart iterative radon function with graded angle steps
            if iter==1
                theta = firstiter;
            else
                thetastep = thetastep/2;
                theta = (-3*thetastep+curangle):thetastep*2:(3*thetastep+curangle);
            end
            theta = mod(theta+90,180)-90; % ensures angle range of [-90,+90)
            R = radon(curimgseg,theta); % Eq. 7
            R(R==0) = nan; % avoids influence of non-participant pixels
            curvar = nanvar(R);
            alltheta = [alltheta theta];
            allvar = [allvar curvar];
            [Rvarmaxval,Rvarmaxin] = max(curvar);
            if Rvarmaxval>curvarmax
                curangle = theta(Rvarmaxin); % Eq. 8
                curvarmax = Rvarmaxval;
            end
            if irl==1 && thetastep<1 % angle resolution less than 1 deg
                irl=2; % iterative radon level 2, where step-size is decided for given dv/v
                curmpa = abs(atand((dvov+1)*tand(curangle))-curangle); % Eq. 17
                ws = min(wi,ceil(hi*abs(tand(curangle)))); % Eq. 14
                hs = min(hi,ceil(wi*abs(cotd(curangle)))); % Eq. 14
                ns = floor(wi*delx/ds)*(hs==hi)+((hi*delx*ws)/(ds*hs))*(ws==wi); % Eq. 13
                dels1 = abs(atand(ws/hs)-atand((ws-1)/hs)*(ws>hs)-atand(ws/(hs-1))*(ws<=hs)); % Eq. 12
                deln = dels1/ns; % Eq. 11

            end
            if irl>1 && thetastep<deln % Eq. 11
                ws = min(wi,ceil(hi*abs(tand(curangle)))); % Eq. 14
                hs = min(hi,ceil(wi*abs(cotd(curangle)))); % Eq. 14
                ns = floor(wi*delx/ds)*(hs==hi)+((hi*delx*ws)/(ds*hs))*(ws==wi); % Eq. 13
                dels1 = abs(atand(ws/hs)-atand((ws-1)/hs)*(ws>hs)-atand(ws/(hs-1))*(ws<=hs)); % Eq. 12
                deln = dels1/ns; % Eq. 11
                if thetastep<deln
                    break
                end
            end

            if irl>1 && thetastep<curmpa % Eq. 17
                % actual dv/v calculation
                actdvovper = abs(tand(thetastep+curangle)/tand(curangle)-1)*100; % Eq. 16
                if dvov>actdvovper/100
                    break
                else
                    irl = irl+1;
                    curmpa = atand((dvov+1)*tand(abs(curangle)))-abs(curangle); % Eq. 17
                end
            end
        end
        angle(jj,1,ii) = curangle;

        % actual dv/v
        actdvovper = abs(tand(thetastep+curangle)/tand(curangle)-1)*100; % Eq. 16
        % deln
        ws = min(wi,ceil(hi*abs(tand(curangle)))); % Eq. 14
        hs = min(hi,ceil(wi*abs(cotd(curangle)))); % Eq. 14
        ns = floor(wi*delx/ds)*(hs==hi)+((hi*delx*ws)/(ds*hs))*(ws==wi); % Eq. 13
        dels1 = abs(atand(ws/hs)-atand((ws-1)/hs)*(ws>hs)-atand(ws/(hs-1))*(ws<=hs)); % Eq. 12
        deln = dels1/ns; % Eq. 11

        angle(jj,2:9,ii) = [thetastep segstart(jj)+hi/2 dels1 deln actdvovper iter irl tand(curangle)*delx/delt];

        % unique angles used for radon and variance measured
        [utheta{jj,ii},um] = sort(alltheta);
        uvar{jj,ii} = allvar(um);
    end
    if showimg
        figure(1496);
        % linescan plot with angle
        subplot(1,imgsegsz(3)*2,ii+imgsegsz(3));
        imagesc(imgseg(:,:,ii)); axis image; title(curimtitle{ii});
        set(gca,'XTickLabel',[],'YTickLabel',[]);
        hold on;
        for jj=1:segn
        [xp,yp] = pol2cart(mod(angle(jj,1,ii)*pi/180-pi/2,pi),wi/2);
        line(xrange(1)+wi/2+[-xp xp],angle(jj,3,ii)-[-yp yp],...
            'Color','black','LineWidth',anglineth,'EraseMode','xor');
        end
        hold off;
        if ii==1
            ylabel({'processed image';...
                ['\Deltax = ' num2str(delx) ' \mum/pixel, \Deltat = ' num2str(delt) ...
                ' ms/line, h = ' num2str(imgsegsz(1)) ' pixels']});
            xlabel(['w = ' num2str(imgsegsz(2))]);
        end

        % angle plot
        subplot(2,imgsegsz(3)*2,+(1:imgsegsz(3)))
        plot(angle(:,3,ii)*delt,angle(:,1,ii),['-o' curcolor{ii} ]); hold on;
        if ii==imgsegsz(3)
            legend(curimtitle{1:ii},'Location','s');
            xlabel('time (ms)'); ylabel('\theta (^o)'); hold off
            title (imgtitle); ylim([min(angle(:,1,ii))-5 max(angle(:,1,ii))+5])
        end

        % velocity plot
        subplot(2,imgsegsz(3)*2,imgsegsz(3)*2+(1:imgsegsz(3)))
        plot(angle(:,3,ii)*delt,angle(:,9,ii),['-o' curcolor{ii} ]); hold on;
        if ii==imgsegsz(3)
            legend(curimtitle{1:ii},'Location','s');
            xlabel('time (ms)'); ylabel('v (mm/s)'); hold off
            ylim([min(angle(:,9,ii))-1 max(angle(:,9,ii))+1])
        end
    end
end

warning on
```

Published with MATLAB® 7.5
