# Supplementary figures and images for: Improved blood velocity measurements with a hybrid image filtering and iterative Radon transform algorithm
Source: Front Neurosci. 2013 Jun 18;7:106. doi: 10.3389/fnins.2013.00106 (PMC3684769; doi:10.3389/fnins.2013.00106)

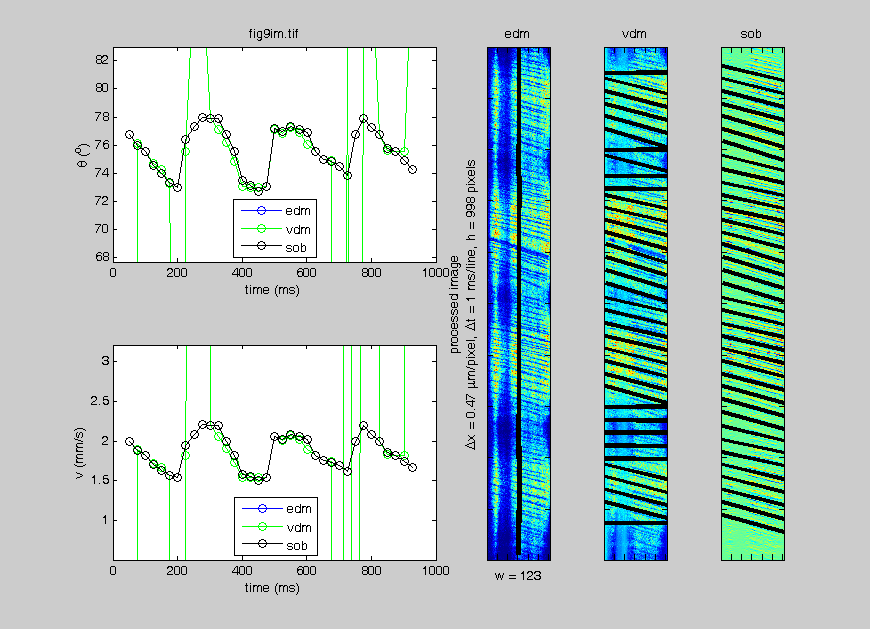

Supplement: Supplementary file 1 [file DataSheet1.ZIP › hybridvelexec_01.png]

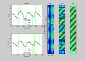

Supplement: Supplementary file 1 [file DataSheet1.ZIP › hybridvelexec.png]

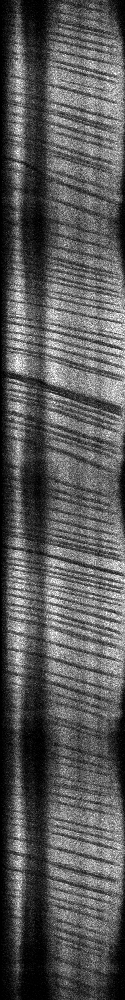

Supplement: Supplementary file 1 [file DataSheet1.ZIP › fig9im.tif]
